# Supplementary material for: Activation of RAAS Signaling Contributes to Hypertension in Aged Hyp Mice
Source: Biomedicines. 2022 Jul 13;10(7):1691. doi: 10.3390/biomedicines10071691 (PMC9313116; doi:10.3390/biomedicines10071691)
Supplement: Supplementary file 1 [file biomedicines-10-01691-s001.zip › biomedicines-1768376-supplementary.pdf]

Supplementary material

Activation of RAAS Signaling Contributes to Hypertension in Aged *Hyp* Mice

Nejla Latic[1], Ana Zupcic[1], Danny Frauenstein[1], Reinhold G. Erben[1*]

*** Corresponding author**:

Reinhold G. Erben, M.D., D.V.M.

Dept. of Biomedical Sciences, University of Veterinary Medicine,

Veterinaerplatz 1, 1210 Vienna, Austria

Phone +43-1-250 77 4550, E-mail Reinhold.Erben@vetmeduni.ac.at

Supplementary Table S1 – Mouse primer sequences for quantitative real-time PCR analysis

**Gene**	**Forward (5´-3´)**	**Reverse (5´-3´)**
Bnp	GCC AGT CTC CAG AGC AAT TCA	GCC ATT TCC TCC GAC TTT TCT
Anp	GGC CAT ATT GGA GCA AAT CCT	GCA GGT TCT TGA AAT CCA TCA GA
